# Mechanism of Repeat-Associated MicroRNAs in Fragile X Syndrome

**DOI:** 10.1155/2012/104796

**Published:** 2012-06-20

**Authors:** Karen Kelley, Shin-Ju E. Chang, Shi-Lung Lin

**Affiliations:** Division of Regenerative Medicine, WJWU & LYNN Institute for Stem Cell Research, 12145 Mora Drive, STE6, Santa Fe Springs, CA 90670, USA

## Abstract

The majority of the human genome is comprised of non-coding DNA, which frequently contains redundant microsatellite-like trinucleotide repeats. Many of these trinucleotide repeats are involved in triplet repeat expansion diseases (TREDs) such as fragile X syndrome (FXS). After transcription, the trinucleotide repeats can fold into RNA hairpins and are further processed by *Dicer* endoribonuclases to form microRNA (miRNA)-like molecules that are capable of triggering targeted gene-silencing effects in the TREDs. However, the function of these repeat-associated miRNAs (ramRNAs) is unclear. To solve this question, we identified the first native ramRNA in FXS and successfully developed a transgenic zebrafish model for studying its function. Our studies showed that ramRNA-induced DNA methylation of the *FMR1* 5′-UTR CGG trinucleotide repeat expansion is responsible for both pathological and neurocognitive characteristics linked to the transcriptional *FMR1* gene inactivation and the deficiency of its protein product FMRP. FMRP deficiency often causes synapse deformity in the neurons essential for cognition and memory activities, while *FMR1* inactivation augments metabotropic glutamate receptor (mGluR)-activated long-term depression (LTD), leading to abnormal neuronal responses in FXS. Using this novel animal model, we may further dissect the etiological mechanisms of TREDs, with the hope of providing insights into new means for therapeutic intervention.

## 1. Introduction

More than 97% of a human genome consists of noncoding DNA, the function of which was unknown until recent years. Variations between individuals' noncoding DNA can sometimes manifest into biological and clinical dysfunction. MicroRNA (miRNA) is a subclass of noncoding RNA that is involved in a wide variety of physiological and developmental events, including developmental timing, embryonic patterning, cell fate determination, cell lineage differentiation, cell proliferation, apoptosis, organogenesis, growth control, and metabolism [1, 2].

MiRNAs are single-stranded molecules consisting of about 18 to 27 ribonucleotides in length and regulate the expression of other protein-coding genes through an intracellular gene silencing mechanism named RNA interference (RNAi). MiRNAs can be located within the noncoding DNA or protein-coding region of DNA [2, 3]. After transcription, instead of being translated the primary miRNA transcript (pri-miRNA) is processed by *Drosha*-like endoribonucleases to a hairpin-like stem-loop precursor, termed “pre-miRNA.” Further processing of the precursor by *Dicer*-like endoribonucleases results in a single-stranded mature miRNA which subsequently forms an RNA-induced silencing complex (RISC) with argonaute proteins and binds complementarily to matched sequences of one or more messenger RNAs (mRNAs) for executing targeted gene silencing through either direct mRNA degradation or translational suppression.

Many introns and untranslated regions (UTRs) of mRNAs also contain tri- or tetranucleotide repeat expansions, capable of being transcribed and processed into repeat-associated microRNAs (ramRNAs) [4–7]. Intronic miRNA is a subset of miRNA that is derived from the noncoding DNA regions of a gene, such as the intron or 5′- and 3′-UTR. In vertebrates, the biogenesis of intronic miRNAs involves five steps [8, 9]. First, miRNA is transcribed—as a long primary precursor microRNA (pri-miRNA)—by type II RNA polymerases (Pol-II) from the intron or UTR of a primary gene transcript [3]. Second, after intron splicing, the long pri-miRNA is excised by spliceosomal components and may be further processed by other *Drosha*-like RNaseIII endonucleases/microprocessors to form precursor microRNA (pre-miRNA) [8–11]. However, intronic miRNA precursors may also bypass *Drosha *processing [12]. During the third step, the pre-miRNA is exported out of the cell nucleus into the cytoplasm, by Ran-GTP and exportin receptors [13, 14]. Fourth, once in the cytoplasm, a *Dicer*-like endoribonuclease cleaves the pre-miRNA to form mature miRNA [9, 10]. Finally, the mature miRNA is assembled into a ribonuclear particle (RNP) to form an RNA-induced silencing complex (RISC) or RNA-induced transcriptional silencing (RITS) complex for executing RNAi-related gene silencing mechanisms [9, 10, 15, 16].

Although the biogenic pathways of small interfering RNA (siRNA)/small hairpin RNA (shRNA) and miRNA are thought to be relatively comparable, many characteristics of the mechanistic components are distinctly different from each other [17, 18]. In zebrafish, we have observed that the stem-loop structure of intronic pre-miRNA is involved in strand selection for mature miRNA during miRNA-associated RISC (miRISC) assembly [10]. Furthermore, unlike the siRNA/shRNA pathway, excessive RNA accumulation can be prevented by the intracellular nonsense-mediated decay (NMD) mechanism, a specific RNA degradation system for unstructured spliceosomal introns [9]. These findings indicate that the siRNA/shRNA pathway is likely lacking some advanced properties required for the regulation of intronic miRNA generation and function.

Given that natural evolution leads to more complex and variable introns in higher animals and plants for the coordination of gene expression volumes and interactions, an intronic repeat expansion or deletion may cause dysregulation of some miRNA biogenesis or miRNA-targeted interactions and thus lead to triplet repeat expansion diseases (TREDs). As shown in Table 1, currently identified TREDs include dentatorubral-pallidoluysian atrophy (DRPLA), fragile X mental retardation syndrome (FXS), Friedreich ataxia (FRDA), Huntington's disease (HD), myotonic dystrophy (DM), spinobulbar muscular atrophy (SBMA), and a number of spinocerebellar ataxias (SCAs). One commonality between these TREDs is that they all express mutant genes with elevated expansion of either CGG/CCG (FXS/FXTAS) or CAT/CTG (others). However, the correlation between the intron-encoded repeat-associated microRNA (ramRNA) and its related TRED remains to be determined. In order to understand the role of a specific ramRNA in the pathogenic mechanism, we must first identify the structure and function of the RNA molecular associated with a TRED. For years the existence of ramRNA has been speculated [5, 7, 19]. Several groups have suggested a correlation between RNA toxicity and TREDs [20–26]; however, there has been no evidence linking a specific ramRNA to a TRED. In this paper, we will describe the process of discovering the first ramRNA identity and how it was used as a tool to establish a transgenic animal model for studying its function *in vivo*.

## 2. Discovery of ramRNAs in FXS

**Figure 1:**
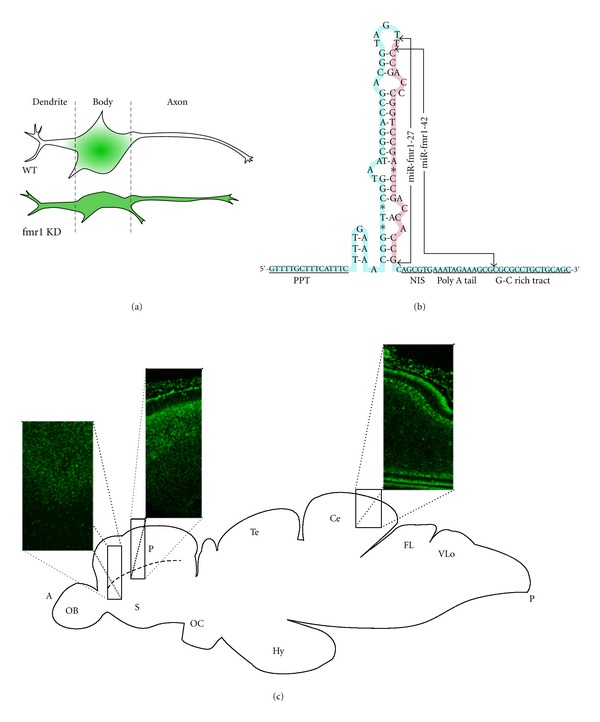
(a) Depiction of the distribution of miR-*fmr1* in wild-type and FXS zebrafish neurons. (b) Sequence diagram of the miR-*fmr1* precursor with both isoforms labeled. Polypyrimidine tract: PPT, nuclear import signal: NIS. (c) Map of wildtype zebrafish brain showing *in situ* hybridization expression patterns of miR-*fmr1* ramRNAs from three sections: (1) cross-section of the lateral pallium, (2) longitudinal section of the pallium-neocortical junction, and (3) longitudinal section of the cerebellum [4].

In 2006, our group successfully found and isolated the first native ramRNA identity, miR-*fmr1*, which is involved in the pathogenetic development of fragile X syndrome (FXS) in a zebrafish model [4, 6]. There are two isoforms of the primary miR-*fmr1 *ramRNAs, miR-*fmr1-27 *and miR-*fmr1-42*, both of which are derived from the *fmr1 *5′-UTR CGG repeat region approximately 65-nucleotide upstream of the translational start codon (accession number NM_152963) (Figure 1(b)) [4]. These two isoforms contain the same seed and core sequence to interact with the zebrafish *fmr1* gene and/or its gene transcripts. Northern blotting of the two miR-*fmr1* isoforms isolated from either the cytoplasm or nucleus of the pallium neurons further demonstrated that miR-*fmr1-42 *is the only ramRNA accumulated in the nucleus of the FXS neurons [4]. Accompanying nuclear miR-*fmr1-42 *accumulation, a significant increase of genomic DNA methylation in the *fmr1 *5′-promoter upstream region was also identified using bisulfite sequencing assays [4]. FXS-related DNA methylation occurs mostly in the CpG-rich binding sites of several *fmr1-*associated transcriptional cofactors, such as NRF1 (GCGCGC), SP1 (GC box), and USF1/USF2 (E box), resulting in transcriptional silencing of the *fmr1* gene. The tissue-specific expression pattern of both miR-*fmr1* ramRNAs in the zebrafish brain has also been identified using fluorescent *in-situ* hybridization (FISH) with a locked nucleic acid (LNA) probe directed against the miR-*fmr1 *seed and core sequence [4, 6]. As shown in Figures 1(a) and 1(c), the normal expression pattern of miR-*fmr1* is limited in the neuronal bodies and nuclei but not the dendrites of the hippocampal-cortical junction, hippocampal stratum radiatum, and cerebellum neurons. In FXS brains, the presence of miR-*fmr1* is however further extended into the dendrites of these neurons and hence causes synaptic deformity. Such broader miR-*fmr1* distribution throughout the dendrites may serve as a marker for FXS diagnosis. 

It should be noted that miR-*fmr1-42 *has a unique pre-miRNA structure consisting of (a) multiple loops and short matched stems in a relatively long hairpin precursor, (b) a nuclear import signal (NIS) motif (probably to allow the reentry of the mature ramRNA into the cell nucleus), and (c) a C/G-rich gene binding motif to recruit DNA methylation machinery (Figure 1(b)) [4]. Deletion of the NIS motif from the miR-*fmr1-42 *precursor has been shown to significantly increase miR-*fmr1* accumulation in the cytoplasm, but not the nucleus of the neurons, suggesting that NIS is responsible for the nuclear entry of miR-*fmr1-42* [4]. These characteristics support a novel disease model in which mature ramRNAs originating from the trinucleotide repeat expansion of a gene can reversely bind back to the corresponding triplet repeat regions of the gene. Individuals with more trinucleotide repeats in the gene generate more mature ramRNAs. As more ramRNAs binding back to the targeted gene, DNA methylation of the triplet repeat regions of the gene occurs, consequently leading to targeted gene inactivation. Due to our discovery of ramRNA and its function in DNA methylation, this ramRNA-induced DNA methylation model may provide further important insights into the mechanism underlying specific gene inactivation in TREDs.

## 3. Correlation between ramRNA and FXS

FXS is one of the most common neuropsychiatric and mental retardation disorders in humans, affecting approximately one in 2000 males and one in 4000 females [27]. In boys, characteristic features of FXS include a long face, prominent ears, large testes, delayed speech, hyperactivity, tactile defensiveness, gross motor delays, and autistic behaviors. Much less is known about girls with FXS. The disease is caused by a dynamic mutation (expansion of microsatellite-like trinucleotide—(cytosine-guanine-guanine)—repeats or termed r(CGG)) at an inherited fragile site on the long arm of the X chromosome, located at the *FMR1 *gene. Due to the dynamic nature of this mutation, trinucleotide repeats can increase in length—and hence in severity—from generation to generation, from person to person, and even within a given person. Patients with FXS have an increased number of r(CGG) > 200 copies in the 5′-UTR of the *FMR1 *gene [20, 28, 29]. The CpG-rich r(CGG) expansion region is often heavily methylated, with a methyl group replacing the hydrogen atom of cytosine (C) and thus the cytosine is conversed to 5-methylcytosine in the *FMR1 *5′-UTR. Such r(CGG) expansion and methylation leads to physical, neurocognitive, and emotional characteristics linked to the *FMR1 *inactivation and the deficiency of its protein product FMRP.

*FMR1* encodes an RNA-binding protein, FMRP, which is associated with polyribosome assembly in an RNP-dependent manner and is capable of suppressing translation through an RNAi-like pathway that is important for neuronal development and plasticity. FMRP also contains a nuclear localization signal (NLS) and a nuclear export signal (NES) for shuttling specific mRNAs between nucleus and cytoplasm [30, 31]. Hence, excessive expression of r(CGG)-derived ramRNAs during embryonic brain development may cause early *FMR1 *gene inactivation, leading to the pathogenesis of FXS. Two theories have been proposed to explain this *FMR1 *inactivation mechanism in FXS. First, Handa et al. [5] found that noncoding RNA transcripts transcribed from the *FMR1 *r(CGG) expansion can fold into RNA hairpins and are further processed by RNaseIII *Dicer* to suppress the *FMR1 *expression. Second, Jin et al. [19] proposed that miRNA-mediated gene methylation may occur in the CpG regions of the *FMR1 *r(CGG) expansion, which are targeted by hairpin RNAs derived from the 3′-end of the *FMR1 *expanded allele transcript. Conceivably, the *Dicer*-processed hairpin RNAs may trigger the formation of RITS assembly on the homologous r(CGG) sequences and result in transcriptional repression of the *FMR1 *chromatin locus; yet, the real mechanism was unclear at that time.

## 4. Vector-Based ramRNA Expression System

**Figure 2:**
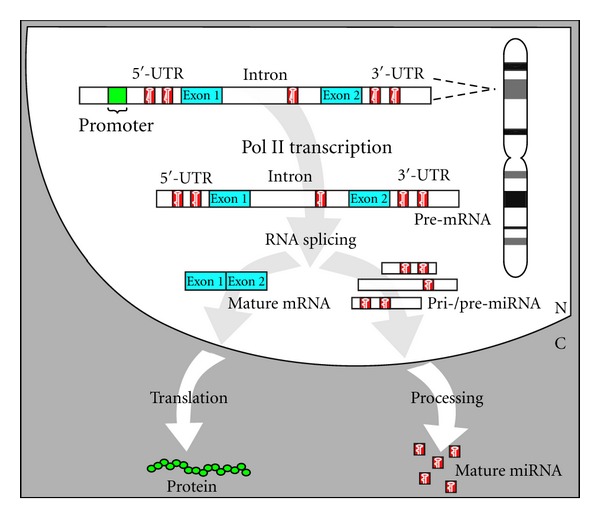
Schematic representation of the mechanism of intronic miRNA expression. After transcription, the miRNA-containing intron is sliced out of the transcript; after further processing by the enzyme *Dicer*, mature miRNA is released and exported out of the nucleus. Meanwhile, the exons are linked together to form mature mRNA which is also transported out of the nucleus for translation.

Ongoing neuroscience research on FXS using animal models (such as the *FMR1*-deleted mouse and fly) has provided a wealth of information in subcellular, cellular, and intercellular networks to delineate the neurobiology of this disorder. Still none of these models demonstrate the pathogenic role of noncoding RNAs in FXS etiology. To overcome this barrier, we have developed and established the first ramRNA-mediated loss-of-*FMR1-*function zebrafish strain as a viable animal model for studying the aforementioned r(CGG)-derived miRNA-induced FXS theory [4, 6, 32, 33]. This novel *in vivo* model may also be used to develop and test drugs or therapies for the treatment of FXS. Our previous studies have shown that effective mature miRNAs can be generated from an artificial intron inserted in a zebrafish vertebrate gene [3, 34]. As demonstrated in Figure 2, the intron containing pre-miRNA structures is cotranscribed with its encoding gene by a type-II RNA polymerase (Pol-II) and further excised by spliceosomal components to form mature miRNAs. Because this intronic miRNA biogenesis pathway is coordinately regulated by intracellular Pol-II transcription, RNA splicing, and NMD mechanisms, the resulting miRNA effector is safe, effective, and powerful as a new genetic tool for regulating targeted gene function [8, 9, 33]. Using this Pol-II-mediated intronic miRNA expression system, we observed target-specific RNAi effects of various man-made miRNAs in mouse and human cell lines *in vitro *[3, 33, 35] as well as mouse skin, chicken embryo, and zebrafish *in vivo* [4, 6, 9, 32]. Based on similar expression designs, Zhou et al. [36] and Chung et al. [37] have also observed that both native intergenic and intronic miRNAs possess the same RNAi effectiveness, while the use of intronic miRNA allows coexpression of a protein marker with the miRNA at a defined expression ratio. Given that there are currently over 1000 native miRNA species found in vertebrates and many more new miRNA homologs continue to be identified, we are able to utilize this intronic miRNA expression system as a transgenic tool for generating target-specific loss-of-gene-function animal strains or cell lines for evaluating the gene function of interest.

Previously, several kinds of vector-based RNAi systems have been developed based on a directly exonic shRNA expression mechanism, using type-III RNA polymerases (Pol-III) [38–40]. Although some of these studies have succeeded in maintaining constant gene silencing effects* in vivo* [41, 42], they failed to provide tissue-specific RNAi efficacy in a cell population due to the ubiquitous existence of Pol-III activities. Moreover, because Pol-III machinery often reads through a short DNA template in the absence of proper termination, large double-stranded RNA (dsRNA) products (e.g., >30 base pairs) may be synthesized and cause unexpected interferon cytotoxicity, particularly in the vertebrates [43, 44]. Such a problem may also result from the competitive conflict between the Pol-III promoter and another vector promoter (i.e., LTR and CMV promoters). Sledz et al. [45] and Lin and Ying [35] have reported that high concentrates of siRNA/shRNA (e.g., >250 nM in human T cells) can result in strong cytotoxicity similar to that caused by long dsRNAs. Notably, Grimm et al. [46] further demonstrated that the Pol-III-directed RNAi systems often generate high concentrated siRNA/shRNA which can oversaturate the cellular miRNA pathway, resulting in global miRNA inhibition and cell death. In view of these problems, a Pol-II-mediated intronic miRNA expression system has the advantage of its autoregulation by the cellular RNA splicing, and NMD mechanisms [9, 33, 47], both of which degrade excessive RNA accumulation to prevent possible cytotoxicity.

The Pol-II-mediated intronic miRNA expression system is designed around a recombinant gene construct containing one or more splicing-competent RNA introns, namely, SpRNAi [3, 8, 9]. Structurally, the SpRNAi consists of several consensus nucleotide elements such as 5′-splice site, branch-point motif, polypyrimidine tract, and 3′-splice site. A pre-miRNA or pre-miRNA cluster insert is placed within the SpRNAi intron sequence between the 5′-splice site and the branch-point motif. This portion of an intron would normally form a lariat structure during RNA splicing and processing. The spliceosomal U2 and U6 snRNPs, both helicases, may be involved in the unwinding and excision of the lariat RNA fragment into pre-miRNA; however, the detailed processing mechanism remains to be elucidated. Moreover, the SpRNAi contains a multiple translational stop codon motif (Ts codons) in its 3′-proximal region, which, if presented in a premature mRNA, will signal diversion of the premature mRNA processing to the nonsense-mediated decay (NMD) pathway and thus eliminates excess RNA accumulation in the cell. This feature guarantees the safety of the intronic miRNA biogenesis pathway.

Using this intronic miRNA expression construct, we have tested various hairpin-like miRNA precursors (pre-miRNAs), many of which resulted in mature miRNAs with full capacity for triggering RNAi-associated gene silencing effects in mouse, rat, and human cell lines *in vitro* [3, 33, 35] and in mouse, chicken, and zebrafish *in vivo* [4, 6, 10, 32]. Further advances in the intronic miRNA expression system have also been reported in mice; Chung et al. [37] successfully performed ectopic expression of a cluster of polycistronic miRNAs, which were processed into multiple miRNAs via the cellular miRNA pathway. This kind of Pol-II-driven miRNA expression has several advantages over the conventional Pol-III-directed siRNA/shRNA expression systems. First, Pol-II expression can be tissue specific, whereas Pol-III expression cannot. Second, Pol-II expression is compatible with the native miRNA pathway, while Grimm et al. [46] have reported some incompatibility in the Pol-III-directed siRNA/shRNA expression systems. Third, excessive RNA accumulation and cytotoxicity can be prevented by the NMD mechanism of a cellular intronic expression system, but not a direct expression system [46, 48]. Finally, one Pol-II is able to express a large cluster (>10 kb) of polycistronic shRNAs/miRNAs, which can be further excised into multiple shRNAs/miRNAs via the native miRNA pathway, so as to prevent the promoter conflict that often occurs in a vector system containing multiple promoters.

## 5. Transgenic Animal Model of FXS

Animal models mimicking the human developmental events and diseases are essential tools for the advancement of biomedical research. Zebrafish (*Danio rerio*), a fresh water tropical fish, has set an impressive record as an *in vivo *viable model for studies of mechanisms involving in embryogenesis, organogenesis, physiology, and behavior; developmental neuroscience has also benefited from research using the zebrafish model. Advantages of using zebrafish include low cost, easy maintenance, rapid life cycle, small size, and embryonic transparency. Also, zebrafish exhibit fast development (i.e., nervous system precursors presented by 6-7 hour postfertilization (hpf); first neuron formed by 18–24 hpf), large generation number (i.e., clutch sizes from a single mating pair range between 100 to 200 embryos), and the phenotypes can be easily assessed in many high-throughput assays [49–51]. Screening genetic suppressors in zebrafish will advance the understanding of loss-of-gene-function phenotypes that are related to certain diseases and help identify logical drug target candidates. In addition, screening for morphological or behavioral mutants is often more time- and cost-effective than the equivalent assays in mouse. These advantages have provided great advances in understanding the detailed pathological mechanisms underlying brain disorders that may lead to functional and behavioral defects. For example, zebrafish possess three *FMRP*-related genes, *fmr1, fxr1, *and *fxr2* that are orthologous to the human *FMR1, FXR1, *and *FXR2* genes, respectively [52]. The expression patterns of these genes in zebrafish are also consistent with those in mouse and human [52, 53], suggesting that zebrafish is one of the best models for studying human *FMRP*-related disorders.

To investigate the molecular mechanism of r(CGG)-derived ramRNA-mediated *FMR1 *inactivation, we developed a transgenic FXS model in zebrafish, in which fish *fmr1* is silenced by overexpression of an isolated r(CGG) expansion from the *fmr1 *5′-UTR [4, 6]. Our previous reports have demonstrated the use of a pantropic retroviral vector, *pLNCX2-rT*, to deliver a recombinant SpRNAi-containing red fluorescent protein (*SpRNAi-RGFP*) transgene that is able to express desired miRNA precursors (pre-miRNA) in a ubiquitous *actin* promoter-driven green EGFP-expressing Tg(UAS:gfp) strain zebrafish, Tg(*actin*-GAL4:UAS-gfp) [10, 32]. In this FXS model, an isolated *fmr1 *5′-UTR r(CGG) expansion (accession number NW_001511047 from the 124001st to 124121st nucleotide) was incorporated into the pre-miRNA insertion site of the SpRNAi intron. The original weak *fmr1 *promoter (100–1000 copies of mRNA per cell) was further replaced by a fish gamma-aminobutyric acid receptor 2 (*GABA R2*) promoter (5000–15,000 copies of mRNA per cell) to boost the expression of the isolated *fmr1 *5′-UTR r(CGG) expansion. The *pLNCX2-rT* vector was previously modified from a pseudotyped Moloney Murine Leukemia virus, *pLNCX2* (Clontech, Palo Alto, CA), by replacing the original *CMV* promoter with an isolated fish *GABA R2 *promoter and then inserting the *SpRNAi-RGFP *transgene into the gene cloning site of the *pLNCX2* construct, so as to form a transgenic *pGABAR2-rT-SpRNAi-RGFP *retroviral vector. Given that *GABA R2* and *FMR1* genes are closely coexpressed in many major brain areas in particular, cortex, hippocampus and cerebellum [54, 55], the infection of *pGABAR2-rT-SpRNAi-RGFP *retroviral vector generated a novel transgenic zebrafish strain displaying a full spectrum of FXS disorders.

The *pGABAR2-rT-SpRNAi-RGFP *vector so obtained was injected directly into one-cell-stage fertilized eggs or used to prepare high-titer retroviruses for infecting the 1–10 hpf-stage zebrafish embryos [4, 6, 10, 32]. Transgenic F0 zebrafish obtained from this process were selectively separated into four groups based on their different *fmr1 *knockdown levels, as determined by Western blot analysis, including <50%, 50%–75%, 75%–90%, and >90% knockdown of *fmr1 *expression. The zebrafish showing above 90% *fmr1 *knockdown were too unstable to be raised into a transgenic line. We succeeded in raising zebrafish with 75%–90% *fmr1 *knockdown to sexual maturity. These fish were then crossed with one another to generate the F1 founder line with a stable 75%–85% *fmr1 *knockdown rate. After genotyping and transgene sequencing analyses, the F1 and F2 transgenic lines exhibited two copies of the transgene in a consistent genomic insertion site located in the chromosome 18 close to the 3′-side of the LOC565390 locus region—a region that encodes no gene. We have also showed that fish with >90% *fmr1 *knockdown possess on average 3–5 copies of the transgene located in two to three genomic insertion sites. Concomitant insertion is known to frequently occur in high-titer retroviral infection.

The principle of this loss-of-*fmr1*-function zebrafish model and human FXS is based on the same molecular interaction between the r(CGG)-derived ramRNA and the *FMR1 *5′-UTR r(CGG) expansion. Both mechanisms result in similar pathological defects triggered by ramRNA-mediated *FMR1* inactivation. We found that increasing the expression of *fmr1 *5′-UTR r(CGG) expansion results in a corresponding elevation of miR-*fmr1* concentration over 6-fold in the transgenic zebrafish with 75%–85% *fmr1 *knockdown [4, 6]. Because we only isolated 30% of the whole *fmr1 *5′-UTR r(CGG) expansion region, each transgene—after *GABA R2 *promoter-driven transcription—would approximately create a total 2–4-fold increase in miR-*fmr1* production. As a result, the zebrafish with 75%–85% *fmr1 *knockdown express 6-fold more miR-*fmr1* than the wild-type zebrafish, similar to the difference between human FXS (>200 copies) and normal (<50 copies) r(CGG) expansion expression. During native embryonic development, excessive expression of r(CGG)-derived ramRNAs (over 4 fold) is sufficient to inactivate *FMR1* gene transcription [4, 6]. We also found that both human and fish FXS models present similar pathological abnormalities in synaptic connectivity and neuronal plasticity. Fish with 50%–75% *fmr1 *knockdown may be related to fragile X tremor/ataxia syndrome (FXTAS), which expresses a moderate increase of r(CGG) expansion (~120 copies) and often displays elevated *fmr1 *mRNA but not FMRP protein levels, accordingly.

## 6. Same Abnormalities Observed in Human and Zebrafish FXS

Despite some notable differences in the size, the overall organization of major brain components in zebrafish is highly similar to the human brain [56, 57]. As in other vertebrates, zebrafish possess all of the classical sense modalities such as vision, hearing, olfaction, taste, tactile, balance, and sensory pathways. We have compared the phenotypes of human and zebrafish FXS in detail to provide an informative groundwork for the use of this novel r(CGG)-derived ramRNA-mediated animal model for FXS-related research and drug development. Our previous studies using fluorescent three-dimensional (3D) micrograph have shown abnormal neuron morphology and connectivity in the embryonic brains of the FXS fish, reminiscent of those found in human FXS [4, 6]. In the normal fish lateral pallium (similar to human hippocampal-neocortical junction), wild-type neurons present normal dendrite outlines and are well connected to each other, whereas the *fmr1*-knockdown FXS transgenics exhibit thin, strip-shape neurons, similar to the abnormal dendritic spine neurons in human FXS. Synaptic deformity frequently occurs in the *fmr1*-knockdown neurons, indicating that the functional role of *FMR1* is to maintain neuronal plasticity. Similar alterations in synaptic plasticity have been observed to be a major physiological damage in human FXS, particularly in the hippocampal stratum radiatum, layer IV/V cortex and sometimes cerebellum of severe cases [58–61].

*FMR1 *mRNA is present in dendritic spines and translated in response to activation of the type 1 metabotropic glutamate receptors (mGluR-1) in synaptoneurosomes [62, 63]. The activation of mGluR1 stimulates a phosphorylation cascade, triggering rapid association of some mRNAs with translation machinery near synapses and leading to protein synthesis of the mRNAs [62]. FMRP protein is, however, a translational inhibitor that binds with the mRNA species involved in regulation of microtubule-dependent synapse growth and function, including its own mRNA [20, 64, 65]. Such translational suppression in dendritic spines is though to be crucial for eliminating immature synapses and enhancing synaptic strength during brain development. Changes in spine shape are often coupled to the absence of FMRP function in FXS patients [58]. Thus, an increased density of long, immature dendritic spines found in the *fmr1*-knockdown FXS neurons may provide new insights into the role of FMRP in synaptic maturation and pruning. Based on the present evidence, not only FMRP protein but also miR-*fmr1* ramRNA can modulate the expression of certain neural genes involved in synaptic development and maturation.

In three-month-old male FXS zebrafish, excitatory synapses in slices of the pallium-neocortical junction were found to exhibit diminished long-term potentiation (LTP), as compared with wild-type controls [4]. LTP in hippocampus is a learning-related form of synaptic plasticity and is highly involved in changes found in abnormally shaped dendritic spines [66]. This result observed in *fmr1*-knockdown FXS neurons indicates that deficits in hippocampal-cortical LTP mechanisms likely contribute to cognitive impairments in FXS disorders. On the other hand, postsynaptic stimulation of mGluR increases neural protein synthesis and subsequently triggers internalization of *α*-amino-3-hydroxy-5-methyl-4-isoxazole propionic acid (AMPA) receptors. This process is crucial for the expression of long-term depression (LTD), which refers to a long-lasting decrease in synaptic strength to below the normal baseline level. Given that FMRP is a downstream gene stimulated by mGluR to reversely quench this LTD process, deficiency of FMRP in FXS neurons hence interrupts this feedback regulation mechanism and leads to LTD overamplification. In particular, the pallium neuron LTD is augmented in the absence of *fmr1 *[4], suggesting that exaggerated LTD may be also responsible for aspects of abnormal neuronal responses in FXS, such as autism. This exaggerated LTD, however, can be inhibited by treating the brain slices of the FXS fish with mGluR-specific agonists, such as 3,5-dihydroxyphenyglycine (DHPG). These findings in FXS zebrafish raise a possibility in FXS-associated autism, which is also supported by other evidence that induction of mGluR1-dependent LTD is enhanced in pyramidal cells of the hippocampus in *FMR1*-deleted mice [60]. Thus, altered LTP and LTD in FXS hippocampal neurons may explain how and why such *FMR1 *inactivation hinders the normal learning and cognition process in the brain, which is important for the development of human intelligence quotient (IQ).

## 7. Conclusions

**Figure 3:**
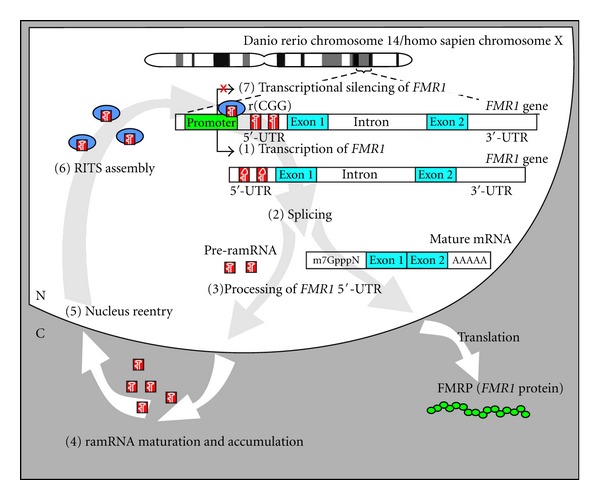
Proposed mechanism of ramRNA-mediated *FMR1 *inactivation in FXS. Fragile X mental retardation 1 (*FMR1*) contains a trinucleotide CGG repeat region (r(CGG)) located in the 5′-UTR of the gene. Expansion of this repeat region of *FMR1* to over 200 copies results in loss of FMRP expression. Based on current data, pathological silencing of FMRP occurs in seven steps. (1) *FMR1*, including the r(CGG) region, is transcribed at an early embryonic stage (day 10 in humans and 12 hours postfertilization in zebrafish). (2) Splicing of the gene transcript to form mature mRNA. During this process r(CGG) molecules are released. (3) The r(CGG) molecules are further processed into repeat-associated miRNA precursors (pre-ramRNA) and exported out of the nucleus. (4) Pre-ramRNA is further processed by the enzyme *Dicer* or a *Dicer*-like endoribonuclease. Mature miR-*FMR1*s accumulate in the cytoplasm near the nucleus. (5) Some miR-*FMR1*s containing a nuclear import signal (NIS) reenter the nucleus by an unknown mechanism. (6) As nuclear miR-*FMR1*s concentrations rise within the nucleus, they may begin to form complexes for RNA-induced transcriptional silencing (RITS). (7) RITS complexes accumulate near *FMR1* promoter and interact with Rad541 and MeCP2, leading to transcriptional silencing of *FMR1 *through a CpG methylation mechanism. Consequently, ramRNA-mediated transcriptional silencing of *FMR1* results in loss of FMRP expression, which is observed in ~99% of patients with FXS.

In sum, our studies have established a novel animal model and possible etiological mechanism for FXS (Figure 3), in which excessive expression of ramRNAs derived from the *FMR1 *5′-UTR r(CGG) expansion results in nuclear ramRNA accumulation and hence inactivated the *FMR1 *gene transcription through promoter DNA methylation. Similar to miRNA biogenesis, *Dicer1* endoribonuclease is required for ramRNA processing. Rad54l and MeCP2 also play a crucial role in the RITS assembly of the ramRNAs responsible for the *FMR1 *promoter methylation. The pathological outcomes of this ramRNA-mediated *FMR1* gene silencing were corresponded to the neurodegenerative and cognitive impairments in FXS disorders, like neuronal deformity, immature synapse formation, long dendritic spine shaping, LTP diminishment, and mGluR-LTD augment. In current studies, we overexpressed one-third of the wild-type *fmr1 *5′-UTR r(CGG) expansion region and found one effective ramRNA, miR-*fmr1-42*; it is estimated that the full *FMR1* r(CGG) expansion in FXS may generate more than 12 kinds of ramRNAs. These findings signify a high similarity between the real human FXS and our ramRNA-induced FXS animal model, which may shed light on new therapeutic interventions.

The list of developmental and degenerative diseases that are caused by expansion of microsatellite-like genomic repeats continues to grow. Many of the trinucleotide repeats are predicted to encode miRNAs; nevertheless, no repeat-associated miRNA (ramRNA) has ever been identified before our studies. We established three important breakthroughs in the understanding of r(CGG)-derived ramRNA function in FXS. First, mature ramRNAs, namely, miR-*fmr1*, can be generated from the 5′-UTR r(CGG) expansion of the *fmr1 *gene in zebrafish, matching the previously predicted epigenetic disease model of human FXS. Second, the ramRNA-induced FXS zebrafish can be raised and maintained to show the same neural defects found in human FXS. Finally, the normal expression pattern of miR-*fmr1* in wild-type zebrafish is limited within the cytoplasm of neuronal bodies, whereas the presence of r(CGG)-associated ramRNAs in FXS neurons can extend into the compartments of nuclei and dendrites, consequently leading to transcriptional *fmr1 *inactivation. These findings confirm the feasibility of using this novel FXS animal model for studying ramRNA-mediated pathogenesis and neuropathology, which may be common in human FXS patients but difficult to identify in other *FMR1-*deleted animal models. In addition, our novel ramRNA overexpression approach may provide further insights into the molecular mechanism of brain-specific trinucleotide repeats for understanding how a ramRNA affects human IQ. Given that there are still many more microsatellite-like nucleotide repeats in the human genome, which may code for a variety of ramRNA species, as might be expected, learning how to use the newly established intronic miRNA expression system for exploiting the functional roles of these ramRNAs *in vivo *will be a forthcoming challenge.

## Figures and Tables

**Table 1:** Triplet repeat expansion diseases (TREDs) that have been identified in humans.

TRED disorders	Site of pathogen	Expansion	Repeat no.
Dentatorubral-pallidoluysian atrophy (DRPLA)	*Atrophin-1*, exons	CAG	49–88
Fragile X syndrome (FXS)	*FMR1*, 5′-UTR	CGG	>200
Fragile X-associated tremor ataxia syndrome (FXTAS)	*FMR1*, 5′-UTR	CGG	55–200
Fragile X syndrome E (FRAXE)	*FMR2*, 5′-UTR	CCG	200–900
Friedreich ataxia (FRDA)	*Frataxin*, intron	GAA	200–1,700
Myotonic dystrophy type 1 (DM1)	*DMPK*, 3′-UTR	CTG	50–1,000
Myotonic dystrophy type 2 (DM2)	ZNF9*, intron 1 *	CCTG	75–11,000
Huntington's disease (HD)	*Huntingtin*, exon 1	CAG	40–121
Huntington's disease-like 2 (HDL2)	*JPH3*, intron, exon, or 3′-UTR	CTG	66–78
Spinobulbar muscular atrophy (SBMA)	*Androgen receptor*, intron	CAG	38–62
Spinal cerebellar ataxia (SCA) types 1–3, 7	*Ataxin 1–3, 7*, exons	CAG	37–300
SCA type 8 (SCA8)	(ncRNA)*UD	CTG	>74
SCA type 17 (SCA17)	*TBP*, exon	CAG	47–63

*UD: undefined.
